# Major depressive episode and postpartum depression: A network analysis comparison on the IGEDEPP cohort

**DOI:** 10.1192/j.eurpsy.2023.2406

**Published:** 2023-05-18

**Authors:** Sarah Tebeka, Christophe Gauld, Raoul Belzeaux, Hugo Peyre, Caroline Dubertret

**Affiliations:** 1 Université Paris Cité, INSERM UMR1266, Institute of Psychiatry and Neurosciences, Team 1, Paris, France; 2 Department of Psychiatry, AP-HP, Louis Mourier Hospital, Colombes, France; 3 Department of Psychopathology of Child and Adolescent Development, Hospices Civils de Lyon, Lyon 1, France; 4 Institut des Sciences Cognitives Marc Jeannerod, UMR 5229 CNRS & Université Claude Bernard Lyon 1, Lyon, France; 5 Aix Marseille Univ, CNRS, Inst Neurosci Timone, Marseille, France; 6 Fondation FondaMental, Créteil, France; 7 Department of Psychiatry, CHU de Montpellier, France; 8 Autism Reference Centre of Languedoc-Roussillon CRA-LR, Excellence Centre for Autism and Neurodevelopmental disorders CeAND, Montpellier University Hospital, MUSE University, France; 9 CESP, INSERM U1178, Centre de recherche en Epidémiologie et Santé des Populations, Villejuif, France

**Keywords:** Major depressive disorder, network analysis, postpartum depression, sadness, suicidal ideations

## Abstract

**Introduction:**

Major depression episode (MDE) and postpartum depression (PPD) have the same diagnosis criteria, but dissimilarities may be present regarding the frequency and structure of depressive symptoms.

**Methods:**

We used data from the IGEDEPP Cohort (France) to examine DSM-5 depressive symptoms in two groups of women: 486 with PPD and 871 with a history of non-perinatal MDE. We compare (i) the frequency of each depressive symptom adjusted for the severity of depression, (ii) the global structure of depressive symptom networks, and (iii) the centrality of each symptom in the two networks.

**Results:**

Women with PPD were significantly more likely to have appetite disturbance, psychomotor symptoms, and fatigue than those with MDE, while sadness, anhedonia, sleep disturbance, and suicidal ideation were significantly less common. There were no significant differences in the global structure of depressive symptoms of MDE and PPD. However, the most central criterion of the MDE network was “Sadness” while it was “Suicidal ideations” for the PPD network. “Sleep” and “Suicidal ideations” criteria were more central for PPD network, whereas “Culpability” was more important for MDE network than for PPD network.

**Conclusion:**

We found differences in depressive symptoms expression between PPD and MDE, which justify continuing to clinically distinguish PPD from MDE.

## Introduction

The distinct nature of postpartum depression (PPD) in comparison to major depressive episode (MDE) is debated: Are they clinically and/or biologically different entities [1–3]? Nowadays, the consensual definition is based on the time of onset, namely an MDE beginning in the postpartum period [4]. However, several definitions coexist depending on the onset time frame considered. The DSM, in its fourth edition, added a specifier “with onset in the postpartum period”, corresponding to an MDE occurring within 4 weeks of birth, thus making PPD a specific MDE for the first time [5]. The DSM-5 has extended this to pregnancy by using “with peripartum onset” [6]. Globally, researchers often adopt a broader definition of postpartum, extending to the first year postpartum [7–9].

Little data exist on the clinical differences between MDE and PPD. It is established that both are heterogeneous disorders in terms of semiology [10–13]. The diagnostic criteria of PPD are those of MDE, and a US population-based study has not been able to distinguish specific symptom profiles based on these criteria [14]. However, some authors suggest that PPD has some specific clinical characteristics. Thus, in the early postpartum period, some studies found that women presented more frequently anxious symptoms, agitation, impaired concentration, and decision-making, or obsessive aggressive thoughts, in particular, harming their newborn, than during non-perinatal depression [15–17]. In addition, physical complaints—headache, fatigue, pain—may also be symptoms reported by postpartum women specifically [18,19]. These analyses are based on models considering each symptom separately, without considering their weight or interaction. Yet network approaches, increasingly used in psychiatric disorders [20], provide a better understanding of psychopathology by considering the combination of symptoms and their interactions [21]. Based on emerging evidence, mood disorders can be conceptualized as causally interacting symptom networks [22–24]. Santos et al. were the first to use this approach for perinatal depression by presenting the symptom network in depression during pregnancy [25]. More recently, Phua et al. compared depressive-anxiety networks during pregnancy and PPD at 3 months postpartum [26]. They found that the central symptoms were different between the prenatal and postpartum depressive-anxiety networks (i.e., feeling worthless or useless in prenatal and feeling overwhelmed or being punished in postpartum), although the network structures remained stable during and after the pregnancy; this suggests that maternal mood and anxiety during pregnancy and during the early postpartum period may have different presentations and etiologies. To our knowledge, there are no previous studies analysing the mutual interactions between PPD symptoms occurring up to 1 year postpartum, nor a comparison between the mutual interactions of MDE symptoms in comparison with the PPD symptoms.

The objective of our work is therefore to describe and compare depressive symptoms in MDE and in PPD the networks of depressive symptoms in MDE and in PPD (i.e., with MDE defined as occurring during the first year after childbirth), and their networks.

## Methods

### Sample and inclusion criteria

Data were extracted from the Interaction of Gene and Environment of Depression during PostPartum Cohort (IGEDEPP, France) composed of 3,310 Caucasian women, French speaking, and were covered by French social insurance. They gave birth in eight maternity departments in the Paris metropolitan area in France, between 2011 and 2016, with follow-up until 1 year postpartum, with an acceptance rate of 61.2%.

Delivery before 32 weeks’ gestation, schizophrenia, or mental retardation were exclusion criteria. Eligible women were given full information about the study by a clinician in the maternity department. Women were included after written consent.

Of the 3310 IGEDEPP women, 3015 (91.1%) were assessed at 8 weeks postpartum and 2351 (71.0%) women were followed up at 1 year postpartum. The women in the IGEDEPP had a mean age of 32 years, and were currently in a relationship (96.7%), employed (93.3%), and had a high level of education (university level or higher) (92.0%) [27]. The research protocol (ClinicalTrials.gov Identifier: NCT01648816), including informed consent procedures, was approved by the French Ethics Committee (Ile de France I) and Data Protection and Freedom of Information Commissions.

### Assessments

Socio-demographic characteristics were assessed at the initial evaluation including age, marital status, educational level, and professional situation.

Each participant was evaluated at three points over the course of 1 year, with a face-to-face standardized and structured interview at the maternity department and with two phone interviews at 8 weeks and 1 year postpartum. All assessments, collecting sociodemographic variables, and the psychiatric history are presented by Tebeka et al. in [27].

These interviews were conducted by clinicians (psychiatrists or psychologists) specifically trained to administer the Diagnostic Interview for Genetic Studies (DIGS) [28], a semi-structured interview.

During the first interview, they collected the psychiatric history, and in particular the history of depression using DSM-5 diagnostic criteria of MDE. In case of MDE history, the perinatal context was assessed by a question: “Did this episode occur during or following a previous pregnancy?”

PPD corresponded to having experienced a PPD between birth and 1 year postpartum: we pooled women with early-onset PPD, assessed during the second interview (at 8 weeks postpartum), and those with late-onset PPD, assessed during the third one (at 1 year postpartum), using DSM-5 diagnostic criteria of MDE [29,30].

In the three steps, the diagnosis of MDE is based on the DSM-5 criteria, that is: (A) the existence of sadness or anhedonia, (B) the co-existence of at least five symptoms during the same 2 weeks period that are a change from previous functioning among sadness, anhedonia, appetite disorders (significant weight loss (without dieting) or gain (change of >5% body weight in a month), or decrease or increase in appetite), sleep disorder (insomnia or hypersomnia), psychomotor symptoms (agitation or retardation), fatigue, culpability, decreased concentration, and suicidal ideations.

For each patient, we also evaluated the severity of the depressive episode (MDE or PPD) via the number of symptoms presented.

For this study, we considered two groups: women with PPD (*N* = 486), and those with a history of MDE with no history of perinatal MDE (*N* = 871).

### Statistical analyses

#### Description of symptoms in PPD and MDE

We have provided the non-adjusted frequency of depressive symptoms of the nine diagnostic criteria for PPD and MDE in a Figure S1. We have provided the frequency of these criteria, for MDE and PPD, as percentages.

In addition, we performed a comparison of these frequencies between PPD and MDE, adjusted for the number of symptoms in each patient, using multiple logistic regression. We provided the odd-ratios and their confidence intervals in a table. This adjusted comparison seems necessary because the difference in overall severity could be related to sample selection (e.g., if patients with PPD have fewer symptoms in terms of frequency, we control for these results based on overall severity, to verify that the difference between the groups does not depend solely on this difference in frequency).

### Network analysis

Symptom networks consist of nodes (symptoms of the DSM-5 MDE) and edges (the connections between the symptoms), which represent the conditional pairwise relations between two symptoms, controlling for all other symptoms in the network. The methodology used in this paper (see The Supplementary Material) is related to the conventional methodology developed in the literature on psychometric analyses in symptom networks [31] and according to the network guidelines for the computational analysis of network properties [32]. Six steps were carried out for this symptom network’s comparative analysis. The first one refers to the network *analysis*; the other two correspond to the network *inferences*; the last three correspond to the analysis of *stability*, the network *comparison*, and the *community* detection.

First, we extracted and labeled symptoms of the DSM-5 diagnostic criteria of the PPD and MDE networks.

Secondly, based on these criteria, we conducted an estimation to build the network graphical representations (Fruchterman–Reingold algorithm) of the PPD network and of the MDE network. The Fruchterman–Reingold algorithm allows that the weight of the connection between two nodes is proportional to the correlation measure, and the place of the node is positioned according to a force-directed graph measure, so that symptoms with stronger and/or more connections are placed closer to each other. A connection between any two diagnostic criteria (symptoms) was computed if these diagnostic criteria were present in the same patient and with nodes depicted closer together more strongly related. Pearson correlations of the dataset were computed, and a matrix of correlation was drawn to show side-by-side the association of two symptoms in the diagnostic criteria of these disorders. As symptoms are binary data (present or absent), we used the Ising Model to mathematically model these pairwise relationships with conditional dependence relations and network regularization [33]. The network regularization is based on the Least Absolute Shrinkage and Selection Operator (LASSO) [34].

Thirdly, we analyzed centrality (local network measures) by quantifying the structure of these two networks (PPD network and MDE network), at a local level, by four metrics of network analysis (Strength: the weighted number of connections for a given node; Closeness: the shortest path length between node; Betweenness: the degree to which a given node acts as a “bridge” connecting different parts of the network; and Expected Influence: the sum of all edges which extend from a given node) [35], also described in The Supplementary Material. Measures of centrality are given with standardized z-scores, that is, standardized coefficients calculated by subtracting the mean and dividing by the standard deviation for each observation. In a nutshell, centrality can be understood to reflect how connected and thus potentially clinically relevant a diagnostic criterion is in a network (via paths through other diagnostic criteria, intervening on a highly central diagnostic criterion, other nodes will be both directly and indirectly affected). In parallel, we give global measures of these networks, related to small-world measures. Small-worldness is measured using clustering coefficient (degree to which nodes in a graph tend to cluster together) and the average shortest path length (the average over the shortest path lengths of all node pairs). Small-world measures for PPD and MDE networks may be used to evaluate the degree of association between criteria in each of these networks. This helps clinicians to rapidly look for the other symptoms of a syndrome (known in network theory as “high signal-propagation speed”), and the global consistency and manipulability of these disorders for clinicians. A network can be called a small world if its index is higher than 1 [36].

Fourthly, because sample size varied across the two networks, the resulting networks would not be comparable due to differential sparsity. To address this concern and to verify the stability of the centrality measures, we analyzed robustness in terms of stability of diagnostic criteria network centralities, with the calculation of the CS-Coefficient. The CS-Coefficient represents the maximum proportion of participants that can be dropped while maintaining 95% probability that the correlation between centrality metrics from the full data set and the subset data are at least 0.70 (see The Supplementary Material for more details).

Fifthly, the two networks were compared to each other with a network comparison tool providing significance indices evaluating the differences between each network. The network comparison tool is a permutation-based hypothesis test named NetworkComparisonTest (NCT), developed by [37].

All these network analysis steps are also described on early- and late-onset PPDs, provided in the Supplementary Material.

All analyses were performed on R (4.1.3). The details of all these computational analyses (extraction and labeling of the symptoms; construction of the symptoms network; computation of the network metrics) are given in the Supplementary Material.

## Results

### Description of sociodemographic characteristics of our groups

The majority of women in our study were between 26 and 34 years old (86.6% in MDE group, 86.8% in PPD group), with an average age of 33 years in both groups. Most of the participants were currently married or in domestic partnership (96.2% in MDE women and 95.9% in PPD group), employed (96.1% in MDE women and 91.6% in PPD group), and had a high level of education (96.3% in MDE women and 90.1% in PPD group).

Among the women included in the PPD group, 45.7% had a personal history of depression.

### Description of symptoms in PPD and MDE

The non-adjusted prevalence of the diagnostic criteria of PPD and MDE is described in Figure S1. The most frequent symptoms in PPD are fatigue (97%), sadness (94%), and anhedonia (83%), while sadness (99%), anhedonia (91%), and fatigue (87%) are the most frequent in the MDE.

The adjustment of the frequencies to the total number of symptoms for each patient is shown in Table 1, with the odd ratios and their confidence interval. Controlling for severity of the disorder, women with PPD were significantly more likely to have appetite disturbance, psychomotor symptoms, and fatigue than those with MDE, while sadness, anhedonia, sleep disturbance, and suicidal ideation were significantly less common.

**Table 1.** Comparisons of symptom frequencies between postpartum depression (PPD) and major depressive episode (MDE) women, after adjusting for disorder severity (via number of symptoms for each participant) (logistic regression: odd-ratio and confidence interval).

	Major depressive episode (MDE)	Postpartum depression (PPD)	MDE vs PPD
	*N* = 865	*N* = 425	
Symptoms	*N* (%)	*N* (%)	Odd-ratio and confidence intervals
Sadness	872 (99)	454 (94)	1.58 [1.35–1.85]
Anhedonia	797 (91)	401 (83)	1.17 [1.08–1.27]
Appetite	285 (32)	258 (53)	0.77 [0. 73–0.81]
Sleep	702 (80)	299 (62)	1.25 [1.18–1.33]
Psychomotricity	579 (66)	394 (81)	0.80 [0.75–0.85]
Fatigue	761 (87)	470 (97)	0.73 [0.67–0.80]
Culpability	686 (78)	348 (72)	1.07 [0.99–1.14]
Concentration	639 (73)	356 (74)	0.97 [0.91–1.03]
Suicidal ideations	295 (34)	50 (10)	1.38 [1.30–1.47]

### Network analysis

**Figure 1.**
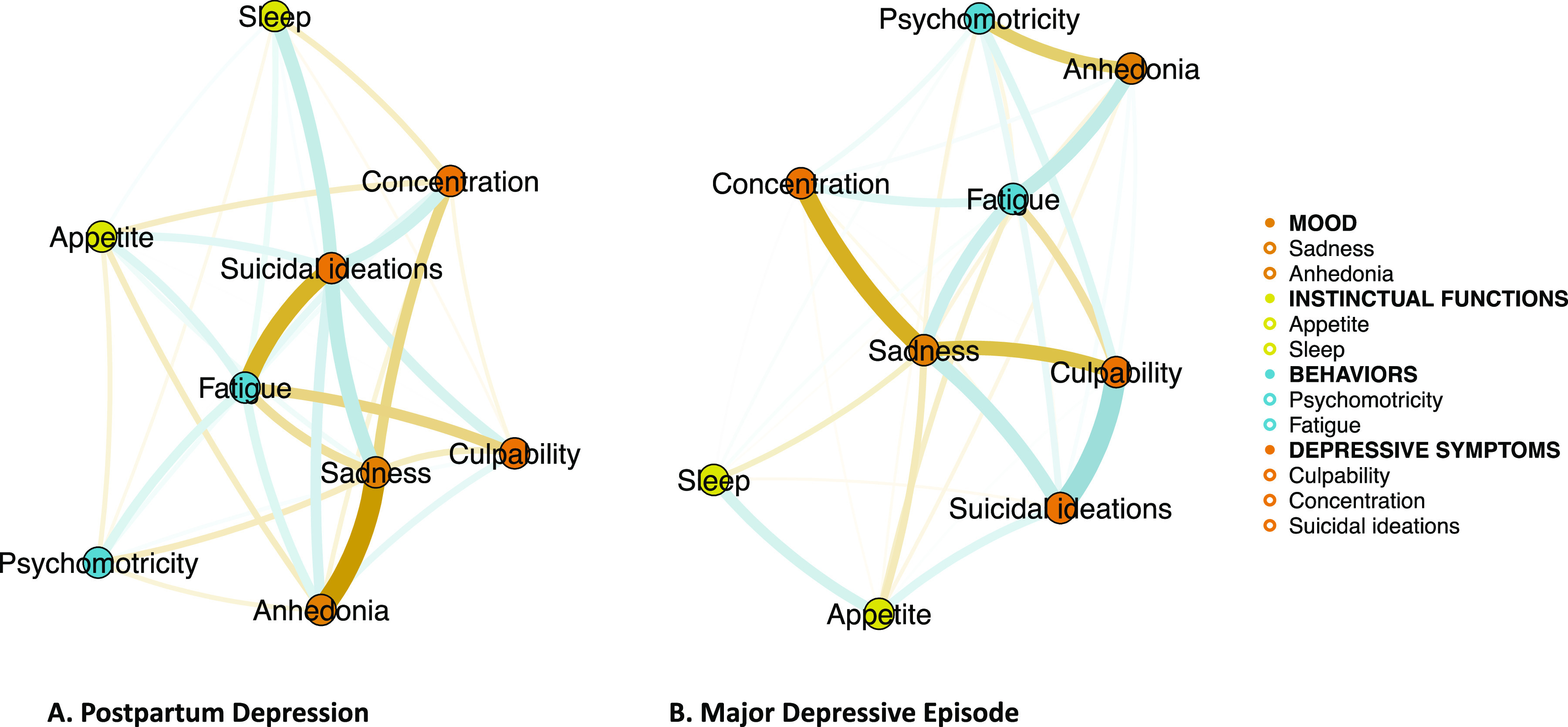
Symptom networks of the diagnostic criteria of a) postpartum depression (PPD) and b) major depressive episode (MDE).Symptom network describes relationships between symptoms (or diagnostic criteria) by drawing a bridge between two diagnostic criteria that are mutually present. The thickness of the lines (edges) represents the level of correlation between the two symptoms. Positive correlations are represented in blue. Negative correlations are represented in yellow. For clarity in the Figure, we have categorized the symptoms into four clinical groups: mood, instinctual functions, behaviors, and depressive symptoms.

Symptom networks of the two DSM-5 diagnostic criteria of PPD and MDE, and topology according to their relationship, are described in Figure 1.

In the PPD network, the highest correlation between two criteria is found between “Sadness” and “Anhedonia” (*r* = −0.46). “Suicidal ideations” is also strongly positively connected with “Sadness” (*r* = 0.31), “Sleep” (*r* = 0.28) and “Concentration” (*r* = 0.24), and strongly negatively connected with “Fatigue” (*r* = −0.37).

In the MDE network, the highest correlation between two criteria is found between “Suicidal ideations” and “Culpability” (*r* = 0.35). “Sadness” is strongly positively connected with “Suicidal ideations” (*r* = 0.22), and strongly negatively connected with “Culpability” (*r* = −0.24) and “Concentration” (*r* = −0.29). “Fatigue” also appears strongly positively connected with “Sadness” (*r* = 0.20) and “Anhedonia” (*r* = 0.24).

Interestingly, the strongest positive connection in both the PPD and MDE networks concerned “Suicidal ideations” and “Sadness” (respectively, *r* = 0.31 and *r* = 0.22).

### Network inferences in the PPD and MDE networks

**Figure 2.**
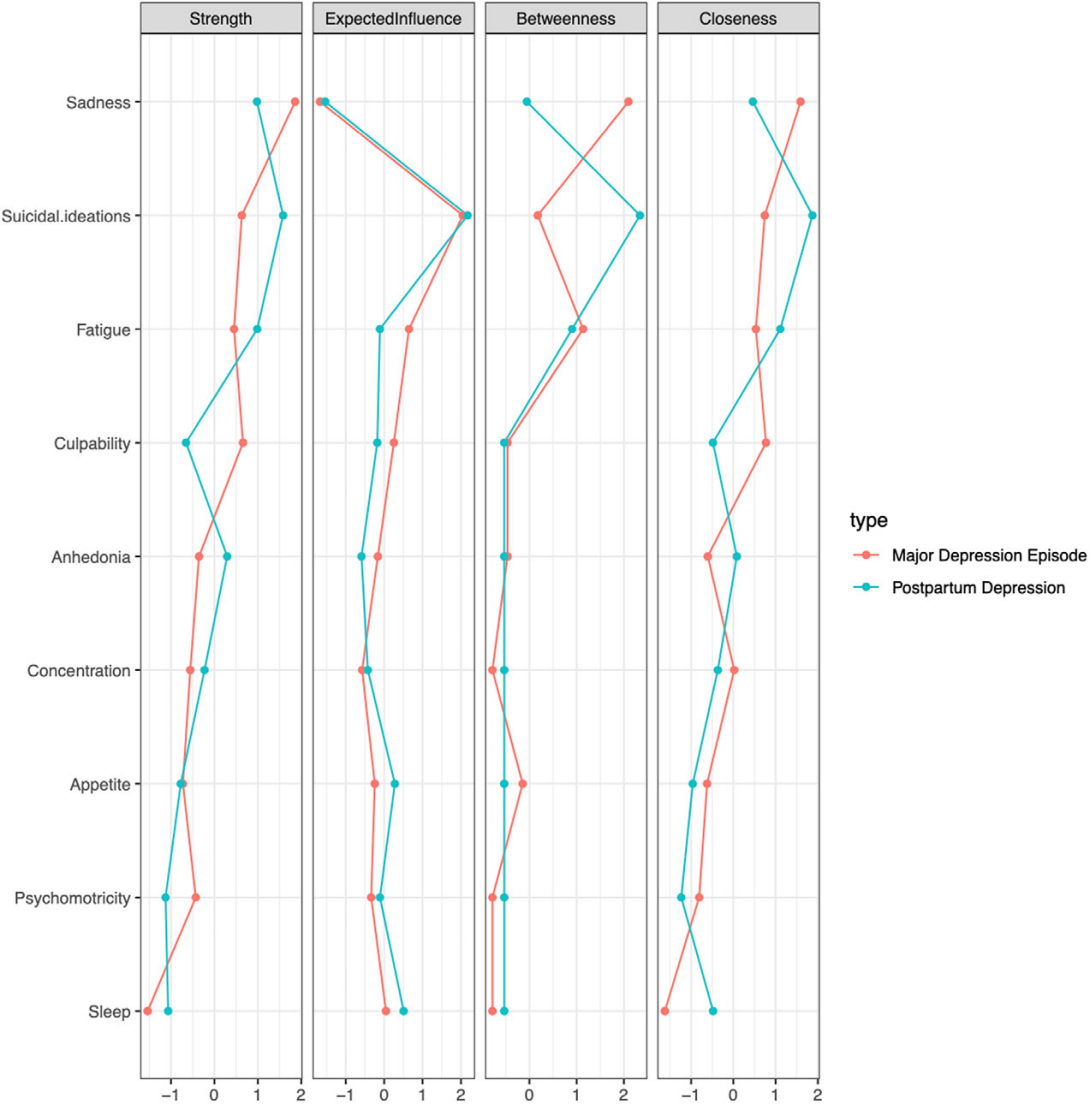
The four measures of centrality (Strength, Closeness, Betweenness, and Expected influence) of major depressive episode (red) and postpartum depression (blue). Each of the four vertical tables corresponds to the two measures of centrality. Within each table, the highest centrality is on the right, the lowest is on the left. Thus, the rightmost criteria are the most central. All the tables are classified according to the decrease in centrality of the Strength (from top to bottom). Centrality numbers at the bottom of each vertical table, on the *x*-axis, show standardized *z*-scores (i.e., standardized coefficients, calculated by subtracting the mean and dividing by the standard deviation for each observation). A *z*-score at −2 on the *x*-axis for expected influence (e.g., Sleep in the Major Depressive Episode) indicates that node has the least expected influence on the network.

Network centrality measures are described in Figure 2. The criterion of “Suicidal ideations” in the PPD constituted the criterion with the highest centrality in the four measures (Strength, Expected Influence, Betweenness, and Closeness), while for MDE “Sadness” was the highest criterion in three of these measures of centrality (Strength, Betweenness and Closeness). This result means that this criterion exhibited a high degree of connections in the entire network (i.e., Strength), an expected important role in the activation, persistence, and remission of the network (i.e., Expected Influence), the shortest path between two random diagnostic criteria of the network on average (i.e., Betweenness), and a shortest mean distance from other criteria (i.e., Closeness).

“Sadness” had the highest Betweenness only for MDE, while “Suicidal ideations” and “Fatigue” had the highest Betweenness for PPD.

Interestingly, “Sleep” and “Suicidal ideations” were more important for PPD than for MDE, whatever the centrality measure used. Conversely, “Culpability” was the only criterion to be more important for MDE than for PPD for all centrality measures and it is strongly connected with “Suicidal Ideations” in the MDE.

Finally, regarding the small-world indices for the PPD network and for the MDE, they were measured at 1.44. Therefore, these networks may be considered as small worlds. The degree of association between criteria in each of these networks was high, allowing clinicians to rapidly look for the other symptoms from a reference symptom collected in the clinical examination. However, the index is not higher for one network than the other, especially because the criteria were similar.

### Network robustness (stability)

In terms of stability, the two networks appeared relatively robust, as shown with the bootstrap analysis (see details in the Supplementary Material, Figure S2).

### Cross-comparisons of networks

General network invariance between the two networks was statistically relevant, as *p*-values were not significant between the two networks of MDE and PPD (*M* = 0.59; *p* = 0.35). In other words, statistically, there is no difference between MDE and PPD networks.

Regarding the centrality (global strength invariance test), no difference was found either (*S* = 0.59; *p* = 0.29). The centrality invariance test was null.

The results and visualization regarding early- and late-onset PPDs are provided in The Supplementary Material (Figure S3). The comparison of the networks shows no statistically significant difference with the NCT, meaning that following a permutation of all the data and calculating the differences for each permutation, there is no difference between the networks.

## Discussion

In this study, both PPD and MDE criteria were described, compared, and analyzed using symptom network analysis. These nine symptoms were evaluated based on the DSM-5 criteria. These symptoms have some stability over classification revisions, as none of them have changed since DSM-III [5,6,38].

The symptom profile was significantly different between PPD and MDE, after controlling for the individual severity (assessed by the number of symptoms). Women with PPD were significantly more likely to have appetite disturbance, psychomotor symptoms, and fatigue than those with MDE, while sadness, anhedonia, sleep disturbance, and suicidal ideation were significantly less common. Agitation has already been described as a specificity of PPD [15,16]. Moreover, a recent meta-analysis confirms a very strong correlation between fatigue and PPD [39]. It is interesting to note that while fatigue is the most frequent symptom of PPD, sleep disturbance is less frequent, suggesting that this fatigue goes beyond sleep disturbance. Fatigue is therefore one of the symptoms that should necessarily be targeted in PPD. Despite a high rate of endorsement of certain criteria (see Table 1 and Supplementary Material (S1), e.g., 99.2% sadness in MDE), we choose not to delete in posthoc some diagnostic criteria, integrated into international classifications and in other network studies (Symptom networks in acute depression across bipolar and major depressive disorders).

Network analysis allows us to go further and to consider the combination of symptoms and their interactions. Thus, network analysis found qualitative differences in core depressive symptoms justify continuing to clinically distinguish PPD from MDE.

Firstly, the highest correlated criteria are qualitatively different in the PPD and MDE networks. For instance, for the PPD network, the highest correlated criterion is the “Sadness” criterion, negatively correlated with “Anhedonia”; while in the MDE network, it was the “Suicidal ideations” criterion, positively correlated with “Culpability”. The negative correlation between “Sadness and “Anhedonia” is specific to PPD. It can be interpreted as a witness of the heterogeneity of PPD: in terms of severity, onset date, but also of presentation with “anhedonia without sadness” PPD and others “sad without anhedonia” [12,13,27].

Moreover, we find in both networks a strong positive correlation between “Sadness” and “Suicidal ideations”. These relationships between sadness and suicidal ideations are consistent with the centrality of the two networks of PPD and MDE. Indeed, interestingly, we find that “Suicidal ideations” constitutes the most central criterion in the PPD. The presence of this criterion can strongly influence the structure of the network, that is, when it is present, PPD can be strongly different from PPD without suicidal ideations. In practical terms, the presence and the high intensity of suicidal ideation in PPD should make the clinician consider PPD to be particularly specific, compared to other PPD patients without this symptom. It is important to note that the absence of centrality in network analysis does not mean a lack of symptom interest, either diagnostically, prognostically, or therapeutically [33]. Indeed, although suicidal ideation is less present and does not structure the overall clinical picture of MDE compared to PPD in our analysis, it should be actively sought out by clinicians. In contrast, in the MDE, “Sadness” is the criterion that most influences the other criteria of the network.

In the same way, the positive correlation between “Culpability” and “Suicidal ideation” is also found in PPD, although it is less marked than in MDE. Such results illustrate that strong guilt (“Culpability”) should elicit suicidal ideation in a patient with MDE—and vice-versa. Other studies have found an association between suicidal ideation and guilt [40–42]. This should encourage clinicians managing women with MDE or PPD, to look specifically for guilt in the assessment of suicidal risk.

Second, the topology of the PPD and MDE networks does not highlight the same central criteria. As retrieved in a large number of studies, sadness represents the most central symptom in the general structure of the network (i.e., in terms of centrality) for the MDE [23,43–45]. Indeed, sadness is a key symptom for the diagnosis of MDE, being one of the two cardinal (i.e., monothetic) criteria [6]. Moreover, this result is also in line with our previous works, in both French and US general population, where we have shown that sadness was indeed a key symptom, with an excellent sensitivity and specificity for the MDE [46,47]. Conversely, suicidal ideations were most central for PPD. Phua et al. who analyzed network of antenatal and post-partum symptoms of depression and anxiety did not include suicidal ideation criteria [26]. Our result has a major resonance considering the place of suicide as one of the main causes of maternal mortality worldwide [48–51].

Moreover, considering the four measures of centrality, “Sleep” was more central for PPD than for MDE, although it is one of the least central nodes for either network. It is well established that postpartum women have more frequent dysregulation of circadian rhythms, and that women with postpartum sleep disturbance are at an increased risk of developing PPD [52–56].

Finally, despite the qualitative differences in core depressive symptoms discussed so far, the statistical analyses did not find any significant difference in the centrality and structures of the PPD and MDE networks. These similarities confirm that PPD could be considered as a kind of MDE, as already mentioned in the work of Hoertel et al. based on item-response theory in the American population [14].

Some limitations could be noted in the present analysis.

First, the IGEDEPP studied is not a French representative sample of postpartum women: they have in particular a high level of education [27]. However, this is the first study to analyze the PPD symptom network, using a large sample of women, prospectively assessed by trained clinicians. Thus, because of this large sample, we believe that our results may be at least generalizable to a similar clinical population of women in the perinatal period. Moreover, MDE history and PPD are assessed at different times, with different hindsight on the event: thus, the recall bias may be more important in the MDE group (where the episode may be very old) than in the PPD group.

Second, there are methodological limitations to this type of study, particularly related to the use of a single population, the limits of psychometric analysis (allowing only cross-sectional collection) or of the network analysis itself, which, by definition, does not provide a latent variable analysis. For instance, if many variables that overlap too strongly in their semantics are included in a network structure, this may yield inadequate solutions [57]. Such modelling, therefore, requires a judicious choice of the key variables in the system. However, the measure of the robustness of the network, which is here considered as a good robustness, offers the opportunity to control the impact of the number of occurrences of a symptom on the centrality measures [35].

Third, apart from the methodological limitations, these results should be interpreted with great caution. The interpretation of the measures of centrality should be particularly prudent [33], especially in the context of suicidal ideations, which should absolutely be identified: even if the symptoms which are strongly related to suicidal ideations (in our analysis) could be absent for a specific patient, these latter should be at the center of the clinical interview and, if they are present, of the patient care.

In conclusion, this study takes an original look at the distinction between MDE and PPD at the level of symptom frequencies and symptom networks. Differences in core symptoms and symptom-symptom associations suggest that PPD has particularities in presentation and possibly etiology, especially in comparison with the MDE. Our symptom network analysis encourages to consider this methodology as an interesting model to refine the nosology, basing ourselves directly on the symptoms described by the patients and highlighted by the clinicians (and not only on their diagnostic scores), while considering the overlap between related disorders but not similar on a semiological scale. Our results constitute a first step in the research on the mechanisms of PPD, which may allow the development of targeted interventions, in particular on suicidal ideation and sleep.

## Data Availability

The data that support the findings of this study are available from APHP. Restrictions apply to the availability of these data, which were used under license for this study. Data are available from the authors with the permission of APHP.

## Clinical trial registration

ClinicalTrials.gov Identifier: 556 NCT01648816.
